# Editorial: Epigenetic modifications associated with abiotic and biotic stresses in plants: An implication for understanding plant evolution, volume II

**DOI:** 10.3389/fpls.2022.1117063

**Published:** 2023-01-06

**Authors:** Mahmoud W. Yaish, Ramanjulu Sunkar, Junzhong Liu, Serena Varotto

**Affiliations:** ^1^ Department of Biology, College of Sciences, Sultan Qaboos University, Muscat, Oman; ^2^ Department of Biochemistry and Molecular Biology, Oklahoma State University, Stillwater, OK, United States; ^3^ School of Life Sciences, Yunnan University, Kunming, China; ^4^ Department of Agronomy Animal Food Natural Resources and Environment, University of Padua, Legnaro, Italy

**Keywords:** stress, epigenetics, evolution, DNA methylation, histone acetylation, noncoding RNA

In the previous volume on this topic, various scientists highlighted the effect of epigenetic changes and their contribution to evolving tolerant plants. This new topic volume aims at providing readers with updates on this subject and highlights the latest understanding of this topic.

Variations in gene expression patterns are important during plant growth and development as well as in plant responses triggered by environmental stresses (Frye et al., 2018). The changes due to environmental stimuli are coupled with plastic modulations in essential physiological and biochemical processes that help plants tolerate stresses (Villagómez-Aranda et al., 2022). These changes may cause transgenerational epigenetic alterations that may enhance the appearance of new tolerant variants (Mladenov et al., 2021). Together with DNA methylation, posttranslational histone modifications such as histone acetylation/deacetylation, methylation, and ubiquitination, are associated with differential gene expression. Furthermore, microRNA (miRNA) can reduce transcript abundance or prevent mRNA translation (Ramzan et al., 2021). Likewise, long noncoding RNAs can play a role in the modulation of gene expression (Jha et al., 2020). These changes may be inherited, especially if plants are constantly exposed to the same stressful environmental conditions, and are now emerging as important molecular players in plant evolution (Gallusci et al., 2022).

For a long period scientists thought that mutations within the DNA were the only mechanism by which a new variety naturally appeared (Yaish). However, these processes are more complicated and involve various genetic and epigenetic changes. Moreover, there is an interaction between the different epigenetic modifications that can also cause changes in the DNA sequences. For example, small RNA molecules (sRNA) prime DNA methylation through the RNA-directed DNA methylation mechanism (Wassenegger, 2000), and DNA methylation and heterochromatin modifications can modulate the expression, excision, and insertion of the transposable elements (TEs) and thereby enhance plant immunity against pathogens (Quadrana et al., 2019). The integration of the TEs within the genome increases genetic diversity, and consequently, natural selection might favor stress-tolerant plants (reviewed by Hannan Parker et al., 2022). Additionally, the accumulation of DNA methylation over time may lead to changes in chromosomal homologous recombination frequency (HRF) in somatic and, perhaps, in sex cells, which leads to changes in DNA sequence (Kovalchuk, 2021). Therefore, epigenetic modifications and the subsequent DNA variation may explain the natural appearance of new variants through natural selection, which led to rapid evolutionary processes (Habig et al., 2021).

Despite tremendous progress in deciphering the mechanism behind evolution, other factors that control the development and evolution of new variants may exist. Therefore, in-depth research is needed to better understand the underlying molecular mechanisms. For instance, in the current volume, a review article by Zhang et al. highlights the importance of the noncoding RNA species such as sRNA, miRNA, and long noncoding RNAs (lncRNAs) in adaptation to abiotic stresses. Therefore, these noncoding RNAs are proposed to be useful in plant breeding programs aiming at improving plant stress tolerance. Similarly, a review by Nunez-Vazquez et al. reveals a complicated and well-conserved relationship between histone sequence variations and the histone modifications that occurred in specific tissues during development and under various stress conditions. Since abiotic stress could be mimicked empirically, there is a good opportunity to study the epigenetic marks or histone variant alterations in response to abiotic and biotic stresses to better understand the complex relationship between the histone variants and epigenetic modifications.

Histone modifications are often associated with abiotic and biotic stress responses (Yaish et al., 2011; Al-Harrasi et al., 2018). For example, the histone acetyltransferase GsMYST1 enhances salt tolerance in soybean (Feng et al.). Likewise, pathogen infections can trigger local and global epigenetic changes, such as histone modification in response to pathogens. Hence, chromatin-remodeling factors are considered to be involved in plant immunity (reviewed by Kang et al.).

In the future, unveiling the remaining epigenetic secrets at the molecular level will further enrich our understanding of epigenetics-driven evolutionary processes that lead to the generation of stress-adaptive plants.

## Author contributions

All authors listed have made a substantial, direct, and intellectual contribution to the work and approved it for publication.
